# Wildlife Abundance and Diversity as Indicators of Tourism Potential in Northern Botswana

**DOI:** 10.1371/journal.pone.0135595

**Published:** 2015-08-26

**Authors:** Christiaan W. Winterbach, Carolyn Whitesell, Michael J. Somers

**Affiliations:** 1 Centre for Wildlife Management, University of Pretoria, Private Bag X20 Hatfield, Pretoria 0028, South Africa; 2 Tau Consultants (Pty) Ltd, P/Bag 83, Maun, Botswana; 3 Centre for Invasion Biology, University of Pretoria, Private Bag X20 Hatfield, Pretoria 0028, South Africa; U.S. Geological Survey, UNITED STATES

## Abstract

Wildlife tourism can provide economic incentives for conservation. Due to the abundance of wildlife and the presence of charismatic species some areas are better suited to wildlife tourism. Our first objective was to develop criteria based on wildlife abundance and diversity to evaluate tourism potential in the Northern Conservation Zone of Botswana. Secondly we wanted to quantify and compare tourism experiences in areas with high and low tourism potential. We used aerial survey data to estimate wildlife biomass and diversity to determine tourism potential, while data from ground surveys quantified the tourist experience. Areas used for High Paying Low Volume tourism had significantly higher mean wildlife biomass and wildlife diversity than the areas avoided for this type of tourism. Only 22% of the Northern Conservation Zone has intermediate to high tourism potential. The areas with high tourism potential, as determined from the aerial survey data, provided tourists with significantly better wildlife sightings (ground surveys) than the low tourism potential areas. Even Low Paying tourism may not be economically viable in concessions that lack areas with intermediate to high tourism potential. The largest part of the Northern Conservation Zone has low tourism potential, but low tourism potential is not equal to low conservation value. Alternative conservation strategies should be developed to complement the economic incentive provided by wildlife-based tourism in Botswana.

## Introduction

Nature and wildlife-based tourism provides benefits that compensate to some extent for conservation costs at local, national and global scales [1–3]. These costs can be high [4] and local communities are often burdened with most of the indirect conservation costs, including wildlife damage and restrictions on land-use and use of natural resources [1].

The majority of tourists to Africa are interested in seeing abundant wildlife with a strong focus on a few charismatic species like lion *Panthera leo*, leopard *P*. *pardus*, elephant *Loxodonta africana*, buffalo *Syncerus caffer* and rhinoceros (*Ceratototerium simum* and *Diceros bicornis*) [5–8]. Experienced wildlife viewers have a wider interest [3]. Due to the presence of charismatic species and good visibility, some areas are better suited to wildlife tourism than others [6] and can provide a better economic incentive for conservation.

Financial incentives for conservation are especially important in the case of Botswana where 38% of the country is set aside for conservation in the form of protected areas and wildlife management areas [9]. Tourism contributes directly and indirectly to Botswana’s economy [10]: in 2011 the Trade, Hotels and Restaurants sector contributed approximately 15.0% to the Gross Domestic Product (GDP) [11] while tourism provided an estimated 10.6% of all jobs in Botswana[10].

Wildlife-based tourism in Botswana is primarily in the form of High Paying Low Volume (HPLV) tourism which is concentrated in the Okavango Delta and Chobe National Park[10], both located in the Northern Conservation Zone. The Government of Botswana lease exclusive use areas called concessions to operators. HPLV tourism is implemented through limiting the number and capacity of private lodges per concession; most lodges have a capacity of 16 to 24 beds. Low cost tourists are limited to the public camp sites in the national parks and game reserves while the HPLV tourists also use the private lodges [12]. Botswana phased out sport hunting [13], and is promoting photographic tourism to replace it. This raises the question about the potential to expand photographic tourism in the Northern Conservation Zone. The suitability and constraints for photographic tourism in this zone should be considered when reviewing recommendations to increase tourism capacity and to diversify tourism activities in Botswana[10]. The quality of game viewing will influence the willingness of wildlife-based tourists to pay for safaris [3,14]. This in combination with the tourist capacity allocated and the annual rental required will determine the economic viability of high, medium and low budget tourism in different concessions of the Northern Conservation Zone. Increased numbers of tourist can have direct and indirect impacts on ecosystems and cultures of local people that may not be biologically or socially sustainable [15].

Our objectives were to quantify the differences in wildlife abundance and diversity between the subjectively selected sites for HPLV tourism lodges in northern Botswana and the areas with a perceived low tourism potential, and to develop criteria to evaluate the tourism potential based on wildlife abundance and diversity. We quantified and compared the tourism experiences in areas with high and low tourism potential and discuss the implications of the photographic tourism potential as an economic incentive for conservation in northern Botswana.

## Methods

### Ethics statement

The necessary permit was obtained for this field study from the appropriate agency in Botswana, the Ministry of Environment, Wildlife and Tourism, Private Bag B0199, Gaborone, Botswana (permit number OP 46/1 LXVIII (133)). No animals were sacrificed, collected, sampled or handled for this study. The permit approval included a review of the methods and ethics of the proposal.

### Study area

**Fig 1:**
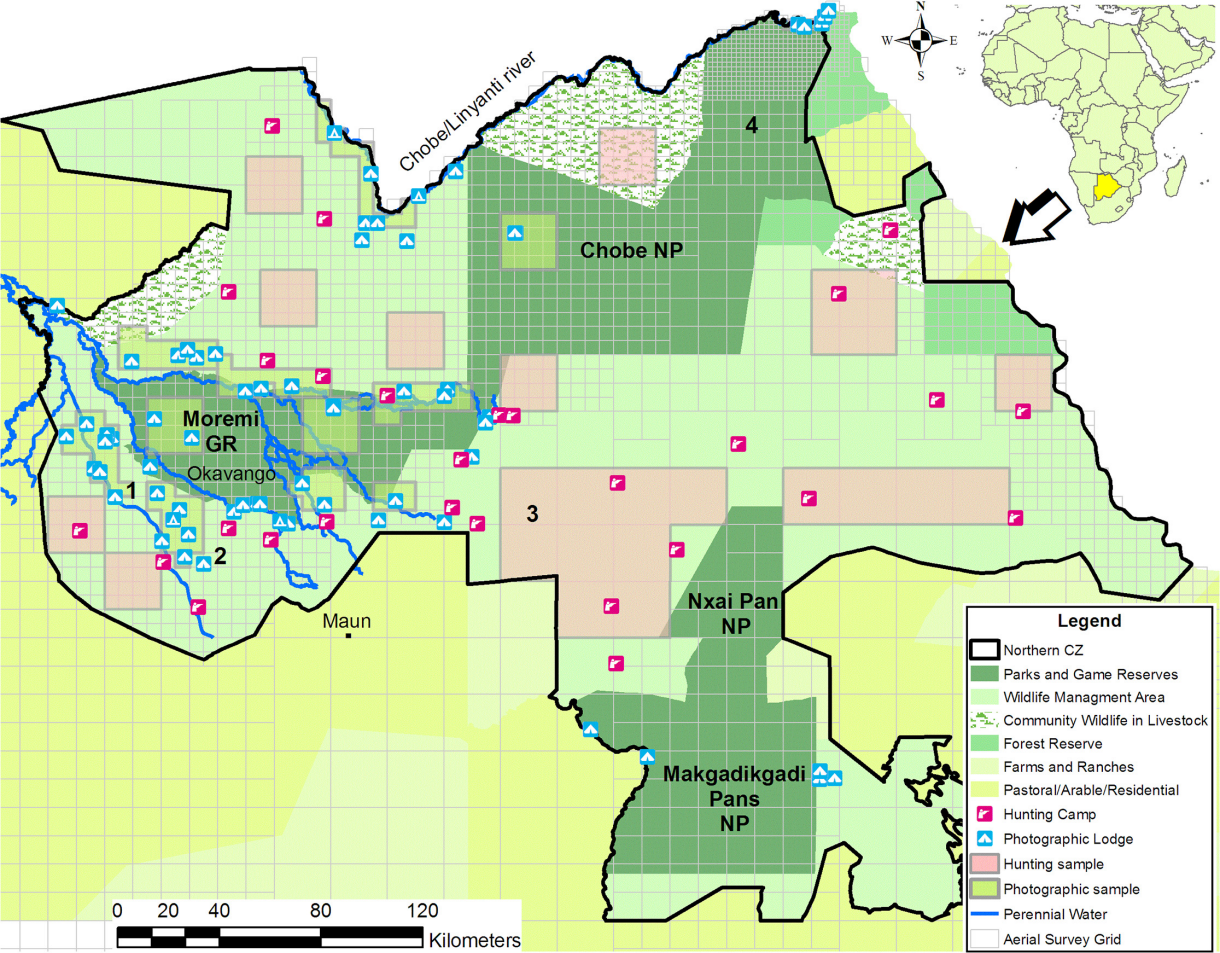
Land use and the locations of photographic and hunting camps in northern Botswana in 2005. Sample areas to compare wildlife biomass and diversity between areas are indicated with a green overlay (High Paying Low Volume photographic tourism and red overlay (without HPLV photographic tourism, used for sport hunting). The four ground survey areas were: 1 = Macatoo, 2 = Xudum, 3 = NG/43, 4 = Nogatsaa.

Botswana is located in southern Africa (Fig 1) and has an area of approximately 582 000 km^2^. The country is relatively flat with a mean altitude of 1000 m above sea-level.

Most of Botswana is arid to semi-arid, with the Kalahari occupying approximately 82% of the country. Rainfall is erratic and ranges from 250 mm per year in the southwest to over 650 mm in the north-east [9]. Over 90% of rainfall occurs in the summer months, between November and April. Apart from the Okavango Delta and the perennial Chobe/Linyanti river system, the only other surface water occurs in rivers and pans during the rainy season**[9]**.

Temperature ranges widely in Botswana. In Maun, located in northern Botswana, the mean maximum daily temperature is 35.5°C in January and the mean minimum is 8.5°C in July. The extreme minimum and maximum temperatures are-6°C and 42°C **[16]**.

Vegetation over most of the country is shrub and tree savanna of the Sandveld. The Hardveld vegetation types are associated with hills and rocky outcrops in the eastern part of Botswana. The Northern Conservation Zone has the wetland of the Okavango Delta, Sandveld, mopane dominated vegetation types and limited Miombo woodland in the north east. The Okavango Delta consists of a mosaic of islands, waterways and seasonal floodplains [9].

Conservation areas comprising of protected areas and wildlife management areas cover 38% of Botswana [9]. The Northern Conservation Zone is an area of 78911 km^2^ and is part of the Kavango-Zambezi Trans frontier Conservation Area (KAZA TFC). The Northern Conservation Zone includes concession areas, forest reserves, Chobe National Park, Moremi Game Reserve, Nxai Pan National Park and Makgadikgadi National Park (Fig 1).

Some concession areas were designated exclusively for photographic safaris while most were multi-use (sport hunting and photographic safaris). The number and capacity of camps and lodges allowed per area were limited. The operators of multiple-use concessions subjectively zoned their areas for photographic and/or sport hunting use, and chose the locations of lodges. Photographic operators deemed parts of the Northern Conservation Zone not suitable for HPLV photographic tourism and thus only camps for sport hunting were established in those concessions.

The Northern Conservation Zone protects key populations of elephant, six of the large African carnivores (lion, leopard, African wild dog *Lycaon pictus*, cheetah *Acinonyx jubatus*, spotted hyena *Crocuta crocuta*, and brown hyaena *Hyaena brunnea*), rare antelope, and a variety of other herbivore species. For this study we considered wildlife as mammals ranging in size from steenbok *Raphicerus campestris* to elephant. This encompassed the three categories that high budget tourists are the most interested in: the big-five, mammals and predators [3]

The quality and diversity of wildlife viewing in the Okavango Delta is a key feature for tourists [14]. The peak tourist season is from July to September, coinciding with the dry season concentration of wildlife along the perennial water sources of the Okavango Delta and Chobe/Linyanti river systems. These are also the two focal areas for the HPLV photographic tourism. Most (44.7%) tourists to Botswana stay for 1–3 three nights while 32.6% stay for 4–7 nights and 19.3% stay for 8 nights or longer [10]. HPLV tourists to private lodges in Botswana stay for 6–8 nights in total, visiting 2–3 lodge for 2–3 nights [12]. On average tourists are able to go on two game drives per day.

Ground transect data were collected as part of a monitoring program in four areas in the Northern Conservation Zone: two areas with high wildlife abundance and two with low wildlife abundance. The Xudum area of concession NG/29 (south-western Okavango Delta) and Macatoo area of concession NG/26 (western Okavango Delta) (Fig 1), are examples of areas with HPLV tourism and high wildlife densities in seasonal floodplain systems with islands.

The Nogatsaa area in Chobe National Park is located approximately 70 km south of the Chobe River (Fig 1). It is a typical dry woodland area that lacks natural perennial water sources. Surface water is limited to seasonal pans and eight artificial water points that are pumped during the dry season. The area is characterised by low wildlife densities during the dry season but is part of the wet season ranges of elephants and buffalo [17].

NG/43 is a dry woodland area southeast of Moremi Game Reserve (Fig 1). It also lacks natural perennial surface water and has a lower long term average rainfall than the Nogatsaa area. Three artificial water points are pumped during the dry season. Wildlife densities are very low during the dry season. Elephants, buffalo, and zebra (*Equus burchellii*) move seasonally into and through the area [17].

### Wildlife abundance and diversity in the Northern Conservation Zone

We used dry season aerial survey data from the Department of Wildlife and National Parks (DWNP) to quantify wildlife abundance and diversity per grid cell in the whole Northern Conservation Zone. Carnivores were excluded from the aerial survey data. The aerial survey data are presented in grid cell format in the Botswana Aerial Survey Information System (BASIS) of DWNP and cover the whole country. The grids vary in size according to the survey stratifications and the resulting spacing of the original aerial transects (1, 3, 6 or 12 minutes) and associated grids (Fig 1) [18].

We calculated wildlife diversity (total number of herbivore species recorded) and mean wildlife biomass (Large Stock Units/ 100 square km) per grid cell for the dry period (1994, 1995, 1996 and 1999 surveys) and the wet period (2001, 2002, 2003, and 2006 surveys) as indicators of wildlife abundance in the Northern Conservation Zone. The dry period was characterized by below average rainfall and low flood levels. This represents conditions at the time that concession holders zoned their concessions and selected sites for camps and lodges. Rainfall and flood levels were higher during the subsequent wet period.

### Wildlife abundance and diversity at sample sites

We compared wildlife abundance and diversity between the subjectively selected sites within the Northern Conservation Zone. The tourism operators selected lodge sites and avoided areas based on their perceived high or low tourism potential. We selected 11 sample sites consisting of 16 grid cells each to represent the main game drive areas used by the HPLV photographic lodges (Fig 1). The sample sites were approximately 400 km^2^, with one exception: the sample site on the Chobe riverfront, where the 16 grid cells covered only 44.44 km^2^ due to the high sampling intensity of the aerial survey. We compared these sample sites with 11 sample sites around hunting camps in areas avoided by the photographic industry (non-HPLV areas) due to the perceived low photographic tourism potential. Similar to the HPLV sample sites we selected 16 grid cells with a total of 400 km^2^ per sample sites. There were three exceptions due to lower sampling intensity of the aerial surveys in parts of the Northern Conservation Zone: one sample site had nine grid cells (900 km^2^); one sample site had four grid cells (1600 km^2^); and one sample site had nine grid cells (3600 km^2^) (Fig 1).

We calculated the mean, standard deviation, and 95% Confidence Intervals of wildlife biomass and wildlife diversity for both HPLV and non-HPLV sample sites. Oneway ANOVAs were used to compare wildlife biomass and diversity between HPLV and non-HPLV cells.

### Tourism potential

We ranked all the grid cells into three classes, High, Intermediate and Low potential for HPLV tourism using criteria based on the 95% confidence intervals for wildlife biomass and species diversity at HPLV and non-HPLV sites. Tourism potential: 1 = low, 2 = intermediate, 3 = high. Cut off values derived from the 95% CI for wildlife biomass (18.8–29.28; 2.47–6.35) and species (5.63–6.38; 1.21–1.64) at HPLV and non-HPLV sites respectively.

The same criteria were applied to the aerial survey data from the wet period (2001, 2002, 2003, and 2006) to assess the variability and potential changes in tourism potential as indicated by wildlife abundance and diversity under conditions of average to above average rainfall and increased flood duration and depth.

### Tourism experience

We used herbivore transect count data that we collected during ground surveys to quantify and compare the tourism experience in areas with high and low wildlife abundance and diversity. Data were collected in the Xudum seasonal floodplain system in 1997, 1998, and 1999. We collected the Macatoo during 2011, after the subsequent decline of wildlife populations reported in the Okavango Delta [19]. The Nogatsaa and NG/43 data were collected in 1998 and 2011 respectively. Data collection included the cold dry, warm dry, and warm wet seasons.

Based on the length of stay for HPLV tourists in Botswana of 6–8 nights in total, visiting 2–3 lodges for 2–3 nights [12], we determined tourist experience for one game drive, four game drives, ten game drives, or more than ten game drives. The high HPLV tourism potential sites are Macatoo and Xudum, indicated in Fig 1 by numbers 1 and 2 respectively. Numbers 3 and 4 represent the locations of NG/43 and Nogatsaa (Fig 1). The counts were carried out with vehicles, boats, or horses. We recorded species, location and total number of animals within 200 m of the transect line for each observation. The analysis includes observations of herbivores from small antelope to elephant and ostriches (*Struthio camelus*). For each transect we calculated the number of observations per kilometre and number of species per kilometre. The number of observations refers to the number of times animals were seen and not to the total number of animals seen.

All statistics were performed with SPSS 16.0.0 and significance level was set at 0.05. We used an ANOVA to test the null hypothesis that the four sites had equal numbers of observations per kilometre. The data were fourth root transformed to meet the assumption of homoscedasticity (equality of variance), and Tukey HSD post hoc tests were done[20]. We also tested the null hypothesis that each site had equal numbers of species per kilometre. After the data were fourth root transformed, Macatoo, Xudum, and NG/43 data were normally distributed but were still heteroscedastic. Thus the Welch t test and Dunnett’s T3 post hoc test were performed[20].

For each species recorded, we determined the number of kilometres per observation. Random sub samples from the Nogatsaa and NG/43 data were included in the subsequent analysis to ensure that the total distance sampled per area were comparable.

We categorized species according to the number of game drives a tourist would need to take in order to observe each species. We assumed that a game drive lasts three hours at a mean speed of 10 km/h and covers a distance of 30 km. “Common” species were expected to be seen in one game drive, “regular” species were expected to be seen in two to four game drives, “uncommon” species were expected to be seen in five to ten game drives, and “rare” species were expected to be seen in more than ten game drives.

## Results

### Wildlife abundance, diversity and tourism potential

**Fig 2:**
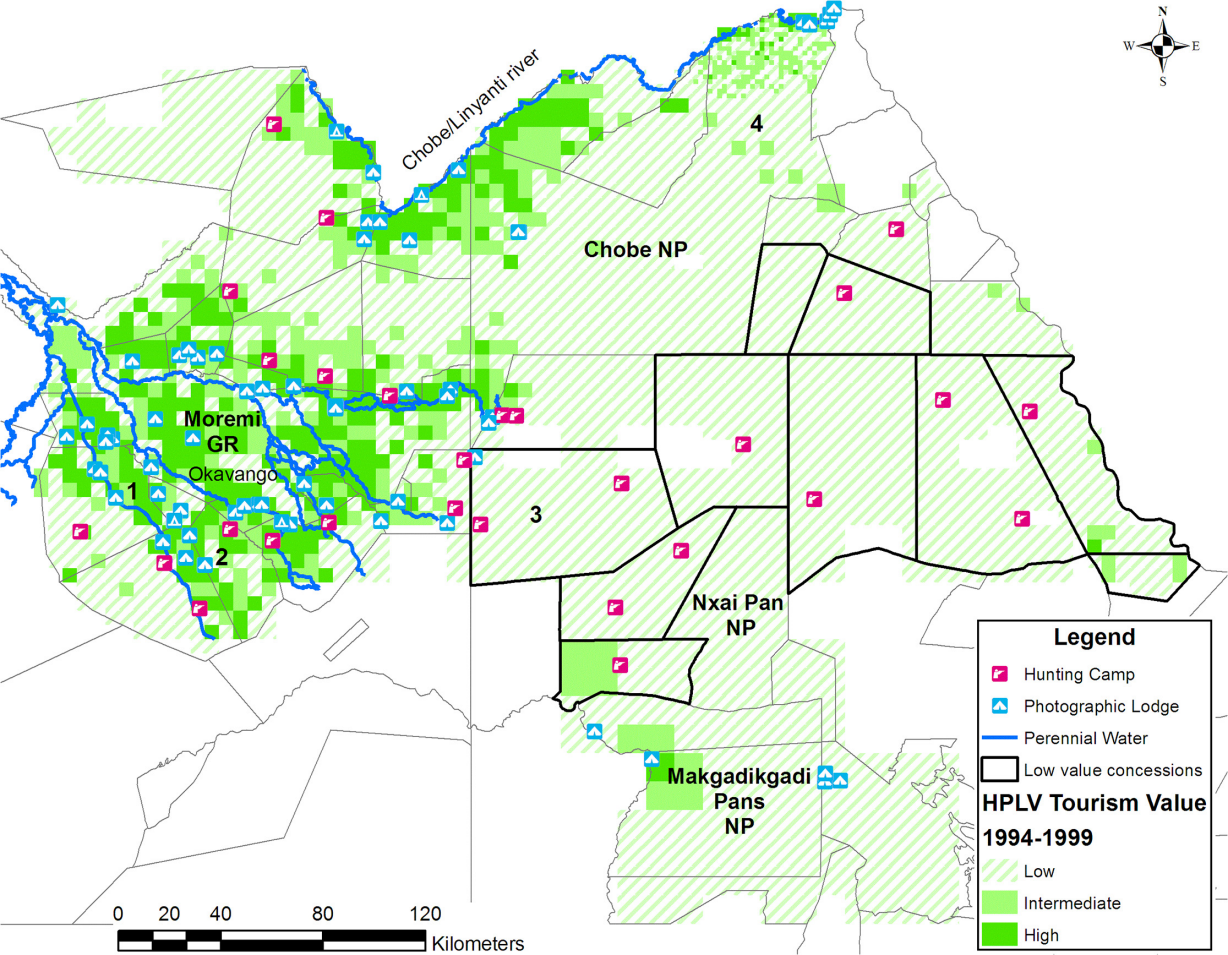
The potential for High Paying Low Volume tourism in the Northern Conservation Zone of Botswana based on wildlife biomass and diversity from 1994 to 1999. The concessions without high tourism potential areas are demarcated by a black boundary. The wildlife biomass and diversity were calculated from the Department of Wildlife and National Parks dry season aerial surveys in 1994, 1995, 1996 and 1999.

**Fig 3:**
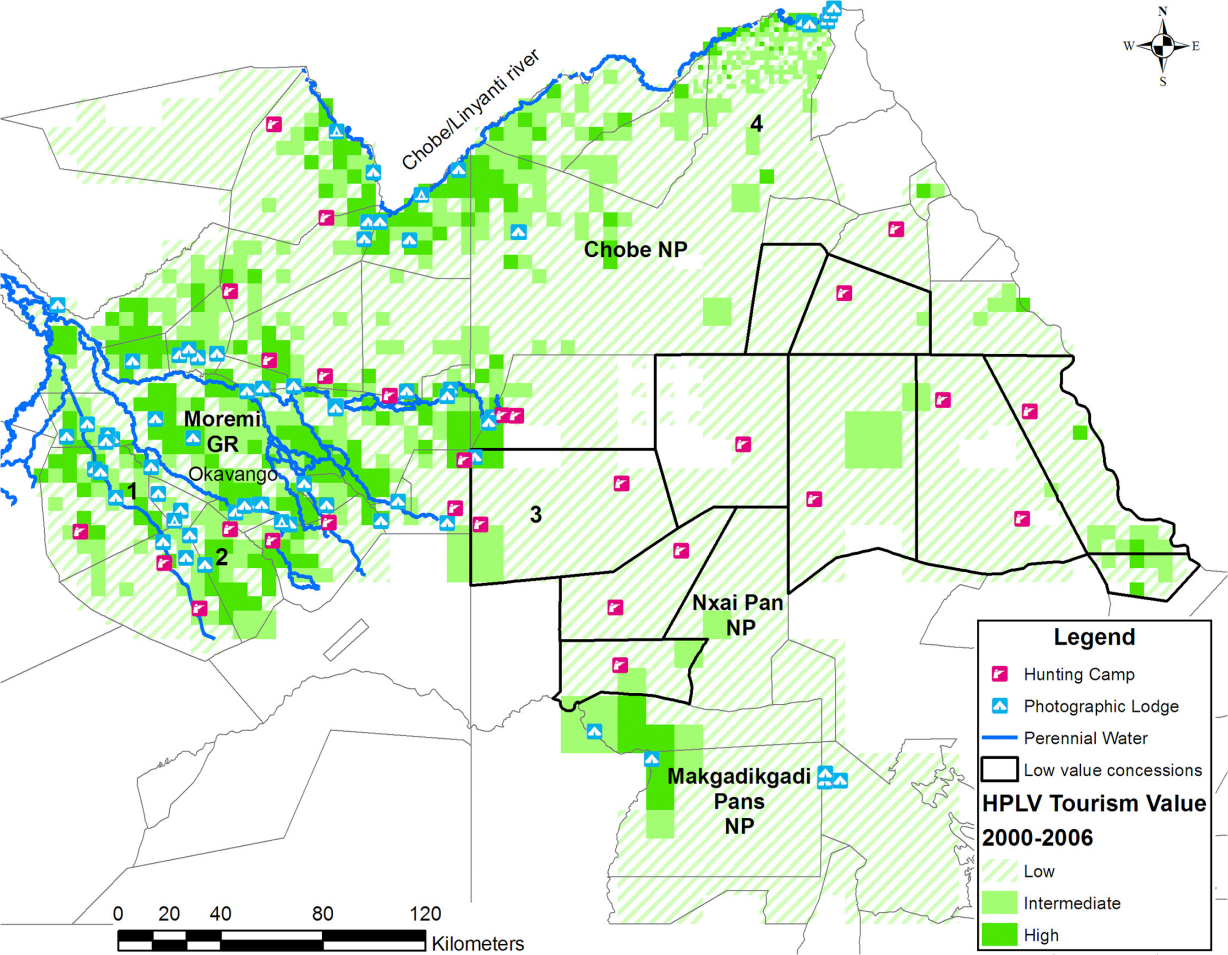
The potential for High Paying Low Volume tourism in the Northern Conservation Zone of Botswana based on wildlife biomass and diversity from 2000 to 2006. The concessions without high tourism potential areas are demarcated by a black boundary. The wildlife biomass and diversity were calculated from the Department of Wildlife and National Parks dry season aerial surveys in 2001, 2002, 2003 and 2006.

We found that the HPLV tourism sample sites in Northern Conservation Zone had a significantly higher mean wildlife biomass (Welch F_1, 258.213_ = 422.797, P = 0.000) and wildlife diversity (Welch F_1, 833.190_ = 47.353, P = 0.000) than non-HPLV tourism sample sites (Table 1) for the 1994, 1995, 1996 and 1999 aerial surveys. This excluded carnivores. Both wildlife diversity (Levene F_1,312_ = 45.009, P = 0.000) and wildlife biomass (Levene F_1,1234_ = 54.988, P = 0.000 were heteroscedastic), therefore equality of means were tested with the robust Welch t test[20]. The wildlife tourism potential of each grid cell was ranked as Low (1), Intermediate (2) or High (3) following the criteria in Table 2. We derived the cut off values in Table 2 from the 95% CI for wildlife biomass (18.8–29.28; 2.47–6.35) and species (5.63–6.38; 1.21–1.64) at the HPLV and non-HPLV sites respectively (S1 File). Fig 2 shows the resulting tourism potential for the Northern Conservation Zone which represents 14% of Botswana. Only 22% of the Northern Conservation Zone has intermediate to high potential for tourism while 78% has low tourism potential (Table 3). Twenty two concessions in Northern Conservation Zone included areas with high tourism potential while ten did not include high potential tourism areas (Fig 2). There were small differences in the proportions and distribution of areas with low and intermediate to high tourism potential between the dry and wet period (Table 3 and Fig 3).

**Table 1:** Mean, standard deviation, and 95% Confidence Intervals for wildlife biomass (Large Stock Units/ 100 km^2^) and wildlife diversity (number of species) for sample sites with HPLV* photographic tourism and without HPLV photographic tourism in the Northern Conservation Zone of Botswana.

Criteria	Cells	n	Mean	s	95% CI
Wildlife Biomass (LSU/ 100km^2^)	No HPLV	576	4.41	23.71	2.47–6.35
	HPLV	660	24.04	68.75	18.8–29.28
Wildlife Diversity (number of species)	No HPLV	150	1.43	1.36	1.21–1.64
	HPLV	164	6.01	2.47	5.63–6.38

Analysis is based on aerial survey data from 1994, 1995, 1996, and 1999.

*High Paying Low Volume

**Table 2:** Ranking criteria based on the wildlife biomass and diversity to evaluate tourism potential of each grid cell in the Northern Conservation Zone of Botswana.

	Wildlife Biomass (Large Stock Units/ 100 km^2^)		
Wildlife Diversity	≤ 6.35	>6.35–<18.8	≥ 18.8
≤ 2 species	1	1	2
3–5 species	1	2	3
≥ 6 species	2	3	3

Tourism potential: 1 = low, 2 = intermediate, 3 = high. Cut off values derived from the 95% CI for wildlife biomass (18.8–29.28; 2.47–6.35) and species (5.63–6.38; 1.21–1.64) at HPLV and non-HPLV sites respectively.

**Table 3:** The size of areas in the Northern Conservation Zone of Botswana with low, intermediate and high tourism potential based on aerial survey data for the dry period (1994, 1995, 1996 and 1999 surveys) and the wet period (2001, 2002, 2003, and 2006 surveys).

Tourism Potential	1994–1999		2001–2007	
	Area (km^2^)	Area (%)	Area (km^2^)	Area (%)
No data available	-	-	876	1
Low (1)	61769	78	59146	75
Intermediate (2)	8574	11	11374	14
High (3)	8568	11	7514	10
Total	78911	100	78911	100

### Tourist experience

We analysed 224 transects covering 4,656.2 km from four sites (Table 4). We recorded the following species: buffalo, duiker (*Sylvicapra grimmia*), eland (*Taurotragus oryx*), elephant, oryx (*Oryx gazella*), giraffe (*Giraffa camelopardalis*), hippopotamus (*Hippopotamus amphibius*), impala (*Aepyceros melampus*), greater kudu (*Tragelaphus strepsiceros*), lechwe (*Kobus leche*), ostrich, reedbuck (*Redunca arundinum*), roan (*Hippotragus equinus*), sable (*Hippotragus niger*), steenbok (*Raphicerus campestris*), tsessebe (*Damaliscus lunatus*), warthog (*Phacochoerus africanus*), waterbuck (*Kobus ellipsiprymnus*), wildebeest (*Connochaetes taurinus*) and zebra.

**Table 4:** The number and length of transects conducted at two HPLV sites and two non-HPLV sites in the Northern Conservation Zone of Botswana, showing the mode of transport used to collect data.

Site	Type	Transport	Transects (n)	Length (km)
Macatoo (2011)	HPLV	Boat	9	102.6
Macatoo (2011)	HPLV	Horse	13	227.7
Macatoo (2011)	HPLV	Vehicle	16	141.3
Xudum (1997–1999)	HPLV	Vehicle	59	481.1
NG/43 (2011)	Non-HPLV	Vehicle	40	928.2
Nogatsaa (1997)	Non-HPLV	Vehicle	87	2775.3
Total			224	4656.2

The mean observations per kilometre and mean species per kilometre had normal distributions after being fourth root transformed. The mean observations per kilometre met the assumption of homoscedasticity (Levene F_3,220_ = 1.799, P = 0.148) but mean species per kilometre were heteroscedastic (Levene F_3,220_ = 5.282, P = 0.002).

The results of the oneway ANOVA showed a significant difference between mean observations per kilometre (fourth root transformed) for the sites (F_3,220_ = 66.650, P<0.001). Tukey post hoc tests (α = 0.05) showed that Xudum and Macatoo were significantly different from each other and from NG/43 and Nogatsaa, but NG/43 and Nogatsaa were not significantly different from each other. The Welch t test showed significant difference between mean number of species per kilometre (fourth root transformed) for the sites (Welch F_3, 89.072_ = 98.579, P<0.001). The two non-HPLV sites, NG43 and Nogatsaa, had significantly lower mean number of species per kilometre than the HPLV sites, but did not differ significantly from each other (Dunnett T3 α = 0.05).

**Fig 4:**
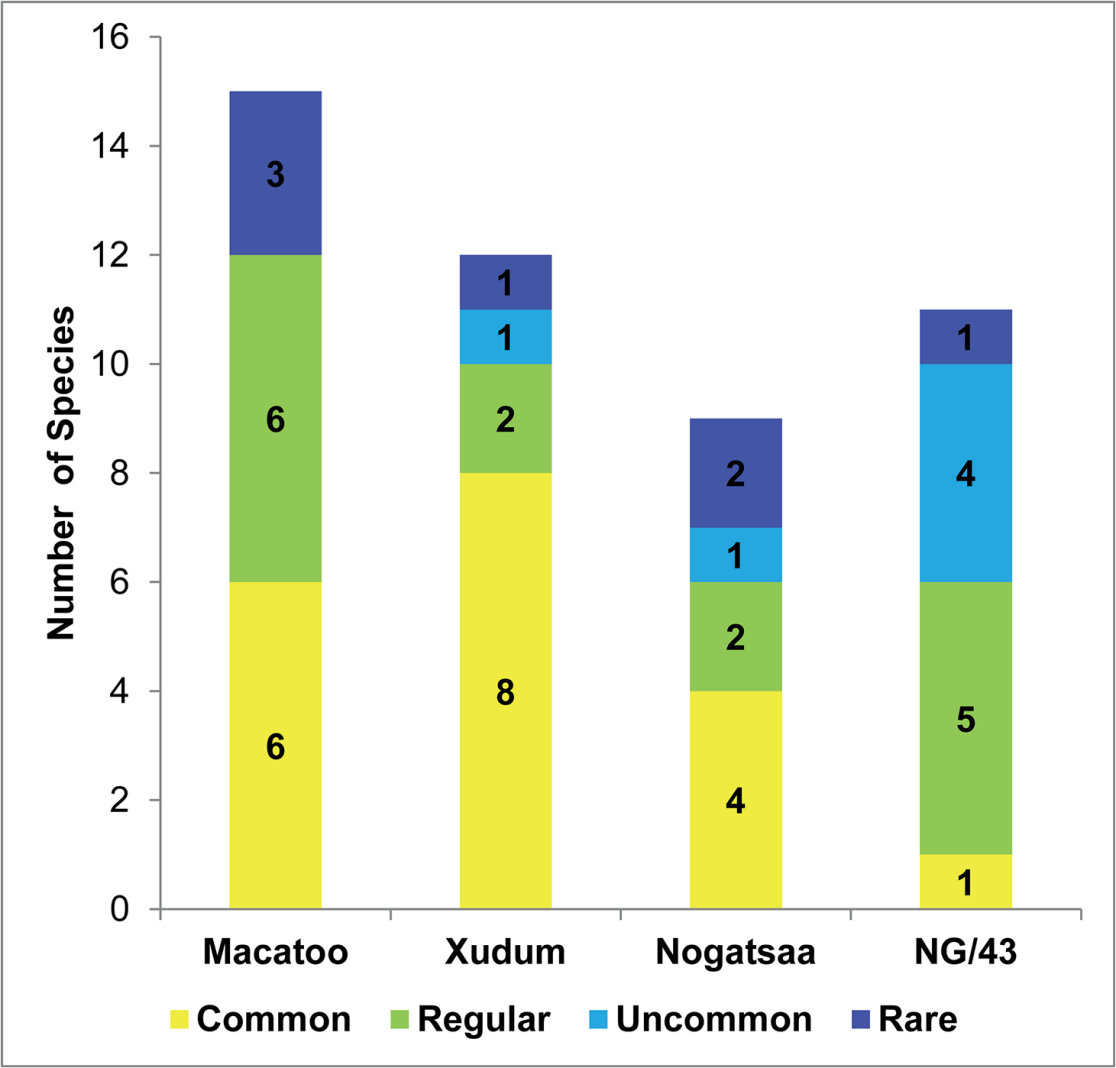
The number of species regarded as common, regular, uncommon and rare at four study sites. Expect to see common species in one game drive, regular species in two to four game drives, uncommon species in five to ten game drives and rare species in more than ten game drives.

The Macatoo and Xudum sites (high tourism potential) had ten to twelve species seen commonly and regularly, more than the five to six species seen at the NG/43 and Nogatsaa sites (low tourism potential) (Fig 4 and Table 5). A tourist on a typical game drive of 3 hours would expect to have 33 wildlife sightings in the Xudum area, one on average every 6 minutes. In contrast a typical game drive in NG/43 should result in 6 wildlife sightings, one every 30 minutes.

**Table 5:** The classification of wildlife species as common, regular, uncommon and rare at four study sites in the Northern Conservation Zone of Botswana and the mean sighting frequency (kilometres/ observation) for each species.

	Macatoo		Xudum		Nogatsaa		NG/43	
	Species	km/obs	Species	km/obs	Species	km/obs	Species	km/obs
Common (1 game drive)	Impala	4.0	Tsessebe	3.4	Elephant	13.0	Steenbok	11.7
	Elephant	12.1	Impala	4.1	Giraffe	20.3		
	Kudu	15.2	Zebra	7.8	Impala	21.1		
	Giraffe	15.7	Giraffe	8.8	Steenbok	23.1		
	Lechwe	15.7	Wildebeest	11.4				
	Tsessebe	18.9	Lechwe	14.9				
			Kudu	15.4				
			Warthog	24.6				
Regular (2–4 game drives)	Warthog	39.3	Steenbok	44.7	Warthog	56.3	Giraffe	43.7
	Zebra	39.3	Reedbuck	61.4	Kudu	101.4	Elephant	47.7
	Buffalo	42.9					Gemsbok	47.7
	Hippopotamus	42.9					Impala	74.9
	Steenbok	42.9					Zebra	87.4
	Reedbuck	78.6						
Uncommon (5–10 game drives)	(none)		Waterbuck	245.8	Sable	253.6	Kudu	131.1
							Duiker	131.1
							Eland	174.8
							Buffalo	262.2
Rare (>10 game drives)	Duiker	471.6	Duiker	491.5	Ostrich	507.1	Warthog	524.4
	Ostrich	471.6			Roan	507.1		
	Wildebeest	471.6						

A game drive is assumed to last three hours and cover up to 30 km.

## Discussion

Applying our tourism potential criteria to the Northern Conservation Zone shows that limited areas have high tourism potential. The data also shows the operators of HPLV lodges have already subjectively selected sites with high tourism potential and avoided areas with low potential. The high potential tourism areas were associated with the perennial water sources of the Okavango Delta, the Kwando/ Linyanti/ Chobe system and the perennial waterholes of the Boteti River in Makgadikgadi National Park. The low tourism potential areas lacked natural perennial water.

Tourism experience as measured by the number of observations and diversity of animals a tourist could expect on a game drive differed significantly between high and low suitability areas. Xudum and Macatoo, which both have high photographic tourism potential, offered tourists an experience with many observations that included a diverse range of species. In contrast, the low potential areas offered very low diversity and frequency of wildlife sightings.

The low potential areas also lacked the charismatic species important to tourists. Elephants and buffalo are present during the wet season but move to the perennial water sources during the dry season [17] which coincides with the peak tourist season. Appropriate data on large carnivores were not available for the analysis, but the expected densities of lion, leopard and spotted hyaena are positively correlated with prey biomass [21] and higher lion densities were recorded in the part of the Northern Conservation Zone where more prey was available [22]. Tourists are more likely to see lion and leopard in the high tourism potential areas than the low potential areas. The following unpublished data (CWW) support this: Lion density in the Okavango Delta is between 5 and 20 times higher than at site 3; Leopard density at site 3 is between 50% and 75% of the density at high tourism potential areas in the Okavango Delta. Two subordinate competitors, cheetah and wild dog, may use areas with low lion and hyaena densities as refuges [23]. Although these two species are expected to occur at higher densities in the low tourism potential areas than in the high potential areas, low visibility due to dense vegetation [9,24] limit all wildlife sightings in the low potential areas.

Although “modified high volume—mixed price” tourism has been promoted since the implementation of the Botswana Tourism Master Plan in 2000 [25], tourists to Botswana remain concentrated in Chobe National Park and the Okavango Delta and photographic tourism has not been diversified into other areas [10]. The photographic operators have not been interested in developing lodges in the concessions that lack high tourism potential. The financial viability of the only exception depends on access to Moremi Game Reserve (pers. comm.; Kgori Safaris (Pty) Ltd, P/Bag 146, Maun, Botswana, info@kgorisafaris.com). In personal communications (CWW) the other nine concessionaires indicated that they could not find photographic operators willing to develop photographic tourism in these concessions to complement their sport hunting safaris. All ten of these concessions were conducting sport hunting and the decision by the government to phase out sport hunting will have the biggest negative economic impact on these areas.

Mladenov *et al*. [14] showed tourists rated the quality of wildlife viewing in the Okavango Delta very high and 71% of them were not willing to pay the same price for diminished wildlife viewing elsewhere. Due to the expectations of high paying tourists, it is not a viable option to divert them to low tourism potential areas. Targeting experienced tourists with wider interests than just the charismatic species, and low budget tourists is an option. Lindsey *et al*.[3] found that low-budget tourists in South Africa were more interested in birds, plants and scenery in contrast to the focus of high budget tourists on the big-five, mammals and predators.

Most of the low tourism potential areas are characterised by large sections of monospecific dense woodland with low visibility and flat terrain lacking focal features [9,24]. The lack of landscape variation combined with the low wildlife numbers and diversity may even be a limiting factor for budget tourism. Low budget tourists rarely used the Nogatsaa area in Chobe National Park during two years of field work in the area (pers. obs.).

We are concerned that concessions with only low tourism potential area have very limited potential to sustain even budget photographic tourists. The Government of Botswana may consider rebates on land rental to entice tourism operators to take the risk to develop new and traditional tourism product in the areas with low tourism potential.

A lack of an economic incentive in the medium to long term may result in pressure to convert the “unproductive area” to non-wildlife land use such as livestock production. Livestock production has a high priority in Botswana: “This deliberate decision was taken to increase our national herd from 2.5 to 3.5 million…….” (Minister of Agriculture, C. De Graaff as quoted in the Ngami Times July 18–25, 2008). Mineral prospecting is taking place in some of these concessions and may impact future land use [24].

Despite the low tourism potential, these concessions have a very high conservation value and form a key area in the Northern Conservation Zone and the larger KAZA TFC [26]. The block of ten concessions forms the only link between the Okavango Delta, Chobe National Park, Nxai Pan/ Makgadikgadi National Park in Botswana and Hwange National Park (to the east) in Zimbabwe. It is critical wet season habitat for the largest remaining elephant population in Africa [19] and a large buffalo population. One of the longest zebra migrations in Africa moves through part of this area [27]. With the low lion density [22] it is also an important refuge area for wild dogs and cheetahs.

Large areas with a mosaic of low and high prey densities are important for the conservation of the African large carnivore guild [28]. The Northern Conservation Zone is such an area and includes significant numbers of lions, leopard, spotted hyaena, cheetah and wild dog [22,29,30]. The northern edge of brown hyaena range falls within the Northern Conservation Zone [31]. Losing part of the ten low tourism potential concessions will impact negatively on the conservation of the large carnivore guild in the KAZA TFC including carnivore species classified by the International Union for Conservation of Nature as ‘Endangered’ (African wild dog), ‘Vulnerable’ (cheetahs and lions) and ‘Lower Risk, Near Threatened’ (leopards and brown hyaenas)[32].

The Government of Botswana has a good conservation track record and there is no immediate threat to these areas. However, pressure from Botswana’s livestock industry for access to areas with limited tourism is likely to increase in the medium to long term to fulfil their aspirations to grow their industry. In the meantime alternative conservation strategies should be developed to complement the economic incentive provided by wildlife-based tourism in Botswana.

## Supporting Information

S1 File Data_hplvtourismareas_northern_Botswana.xlsx. (XLSX)
